# Spatial Transcriptome‐Wide Profiling of Small Cell Lung Cancer Reveals Intra‐Tumoral Molecular and Subtype Heterogeneity

**DOI:** 10.1002/advs.202402716

**Published:** 2024-06-19

**Authors:** Zicheng Zhang, Xujie Sun, Yutao Liu, Yibo Zhang, Zijian Yang, Jiyan Dong, Nan Wang, Jianming Ying, Meng Zhou, Lin Yang

**Affiliations:** ^1^ School of Biomedical Engineering National Clinical Research Center for Ocular Diseases Eye Hospital Wenzhou Medical University Wenzhou 325027 P. R. China; ^2^ Department of Pathology National Cancer Center/National Clinical Research Center for Cancer/Cancer Hospital Chinese Academy of Medical Sciences and Peking Union Medical College Beijing 100021 P. R. China; ^3^ Department of Medical Oncology National Cancer Center/National Clinical Research Center for Cancer/Cancer Hospital Chinese Academy of Medical Sciences and Peking Union Medical College Beijing 100021 P. R. China; ^4^ Cosmos Wisdom Biotech Co. Ltd Building 10th No. 617 Jiner Road Hangzhou 311215 P. R. China

**Keywords:** digital spatial profiling (DSP), intra‐tumoral heterogeneity (ITH), small cell lung cancer (SCLC), spatial transcriptomics

## Abstract

Small cell lung cancer (SCLC) is a highly aggressive malignancy characterized by rapid growth and early metastasis and is susceptible to treatment resistance and recurrence. Understanding the intra‐tumoral spatial heterogeneity in SCLC is crucial for improving patient outcomes and clinically relevant subtyping. In this study, a spatial whole transcriptome‐wide analysis of 25 SCLC patients at sub‐histological resolution using GeoMx Digital Spatial Profiling technology is performed. This analysis deciphered intra‐tumoral multi‐regional heterogeneity, characterized by distinct molecular profiles, biological functions, immune features, and molecular subtypes within spatially localized histological regions. Connections between different transcript‐defined intra‐tumoral phenotypes and their impact on patient survival and therapeutic response are also established. Finally, a gene signature, termed ITHtyper, based on the prevalence of intra‐tumoral heterogeneity levels, which enables patient risk stratification from bulk RNA‐seq profiles is identified. The prognostic value of ITHtyper is rigorously validated in independent multicenter patient cohorts. This study introduces a preliminary tumor‐centric, regionally targeted spatial transcriptome resource that sheds light on previously unexplored intra‐tumoral spatial heterogeneity in SCLC. These findings hold promise to improve tumor reclassification and facilitate the development of personalized treatments for SCLC patients.

## Graphical abstract

Understanding the intra‐tumoral spatial heterogeneity in SCLC is critical for improving patient outcomes and clinically relevant subtyping. Zhang et al. performed a spatial whole transcriptome‐wide analysis of 25 SCLC patients at sub‐histological resolution using GeoMx Digital Spatial Profiling technology to unravel intra‐tumoral multi‐regional heterogeneity. These findings hold promise to improve tumor reclassification and facilitate the development of personalized treatments for SCLC patients.

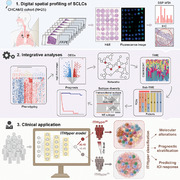

## 1 Introduction

Small cell lung cancer (SCLC) is a highly aggressive and deadly malignancy with dismal survival outcomes. The five‐year overall survival rate is less than 7% for most patients.^[^ ^1^ ^]^ Despite recent advances in treatment, including combinations of immunotherapy and chemotherapy, there has been limited improvement in survival outcomes over the past decades.^[^ ^2^ ^]^ Patients initially respond to these treatments, but quickly develop resistance, leading to rapid disease progression and poor prognosis.^[^ ^3^ ^]^

A primary challenge in current clinical practice for SCLC is the “one fits for all” strategy, which fails to account for inter‐ and intra‐tumoral heterogeneity (ITH), which can significantly influence patient outcomes, response to treatment, and resistance development.^[^ ^4^ ^]^ Although previous studies have investigated inter‐tumoral heterogeneity in SCLC, and defined four major molecular subtypes using conventional bulk RNA sequencing,^[^ ^5^ ^]^ recent advances in single‐cell RNA sequencing (scRNA‐seq) and spatial transcriptomics (ST) have allowed a deeper exploration of intra‐tumoral heterogeneity (ITH).^[^ ^6^ ^]^ In SCLC, scRNA‐seq has begun to reveal ITH‐related molecular profiles within individual tumors, shedding light on the complex cellular ecosystem and heterogeneous characteristics driven by the tumor microenvironment (TME).^[^ ^7^ ^]^ However, scRNA‐seq primarily provides compositional insights into the TME but lacks the structural context of this environment.^[^ ^7a^ ^]^ Understanding intra‐tumoral spatial heterogeneity in SCLC is critical to improve patient outcomes and clinically relevant subtyping. A deeper understanding of the complex ecosystem of the tumor microenvironment would shed light on the underlying ITH characteristics in SCLC. However, current research on ITH is mainly based on mouse models, circulating tumor cells (CTCs), or samples from chemotherapy‐resistant patients with advanced disease using bulk transcriptomics or single‐cell RNA sequencing (scRNA‐seq) approaches, without taking into account the histological composition of the TME.^[^ ^7^, ^8^ ^]^ A critical knowledge gap remains regarding the extent of heterogeneity potentially elucidated through in‐depth molecular profiling at the histopathological level in primary SCLC specimens.

From a clinicopathological perspective, we speculate that the histological structure of sub‐regional ecosystems is closely related to the biological behavior of tumors. To fill this gap, digital spatial profiling (DSP) has emerged as a powerful tool for addressing biological mechanisms in a spatially resolved manner in many cancers. In this study, we perform a comprehensive whole transcriptome atlas of 79 spatially defined regions from 25 SCLC patients at the sub‐histological level using the DSP technology. Our study has generated a preliminary tumor‐centric spatial transcriptional profile that unravels previously unexplored intra‐tumoral spatial heterogeneity in SCLC. This study provides novel molecular insights that may be valuable in developing novel classifications of SCLC and designing effective clinical regimes to improve patient outcomes.

## 2 Results

### 2.1 Digital Spatial Transcriptome Profiling in SCLC

**Figure 1:**
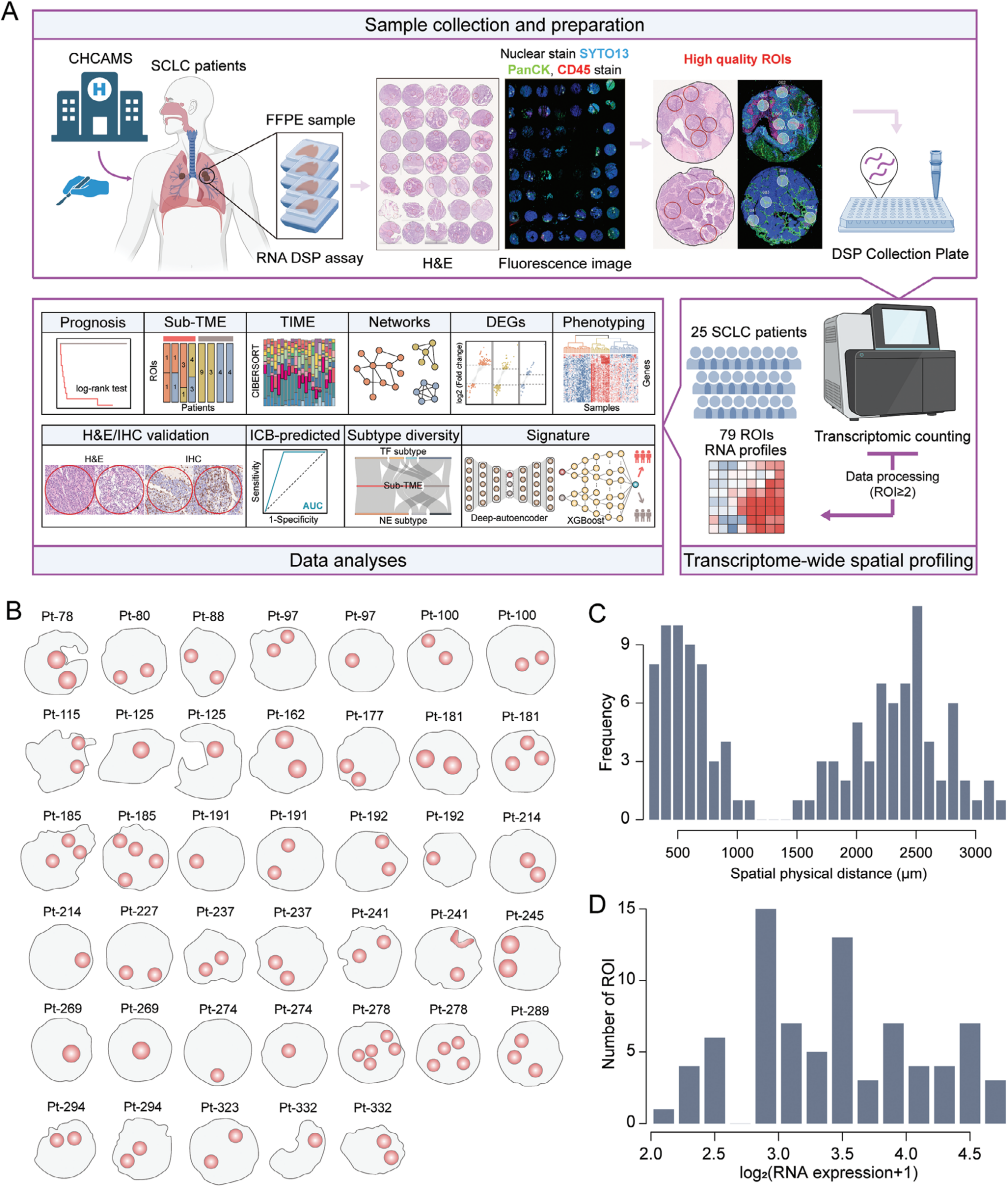
Transcriptome‐wide spatial profiling of SCLCs. A) Schematic of the study workflow. The whole process includes TMA construction, fluorescence antibody incubation, probe hybridization, ROI selection and segmentation, barcode sequencing, DSP data processing and analysis. B) Schematic representation of 79 ROIs from 25 SCLC patients. C) Histogram showing the distribution of pairwise spatial physical distance (SPD) between ROIs. Distance was used with µm. D) Histogram of the number of ROIs with the average expression presented in log format. The average expression was transformed with log_2_(x + 1). DSP, digital spatial profiling; TMA, tissue microarray; ROIs, regions of interest.

To comprehensively explore intra‐tumoral heterogeneity at the spatial transcriptome‐wide scale in SCLC, we used DSP technology with a panel covering over 18,000 protein‐coding transcripts (Whole Transcriptome Atlas, WTA) in situ. We delineated tumor‐immune compartments using fluorescence‐conjugated antibodies (PanCK and CD45) (**Figure** **1**A). A total of 79 tumor‐specific regions of interest (ROIs) were collected from 25 SCLC patients. Due to our stringent selection criteria, the number of ROIs per patient varied from 2 to 9, with a median of 3, based on pathological observation of paired H&E slides on each TMA core (Figure 1B; Figures S1 and S2, Supporting Information).

The spatial physical distance (SPD) between any two ROIs within a single tumor varied from 257 to 3,168 µm, with a median of 1, 843 µm (Figure 1C). High‐throughput sequencing of gene barcodes from ROIs resulted in a median of 6,280,000 aligned reads per ROI, with an interquartile range of 4,080,000 to 10,320,000. Analysis of the density distribution of normalized gene expression across individual ROIs showed decent homogeneity, with no significant bias related to patient, sex, or tumor location, ensuring the reliability and robustness of the data (Figure S3, Supporting Information). We included 18,676 genes from the WTA panel in all subsequent analyses to increase the likelihood of including all genes potentially associated with ITH. The distributions of average gene expression in log‐transformed format for each ROI ranged from 2 to 4.755, generating a normal‐like pattern (Figure 1D).

### 2.2 Spatial Analysis Reveals Intra‐Tumoral Transcriptomic Heterogeneity

**Figure 2:**
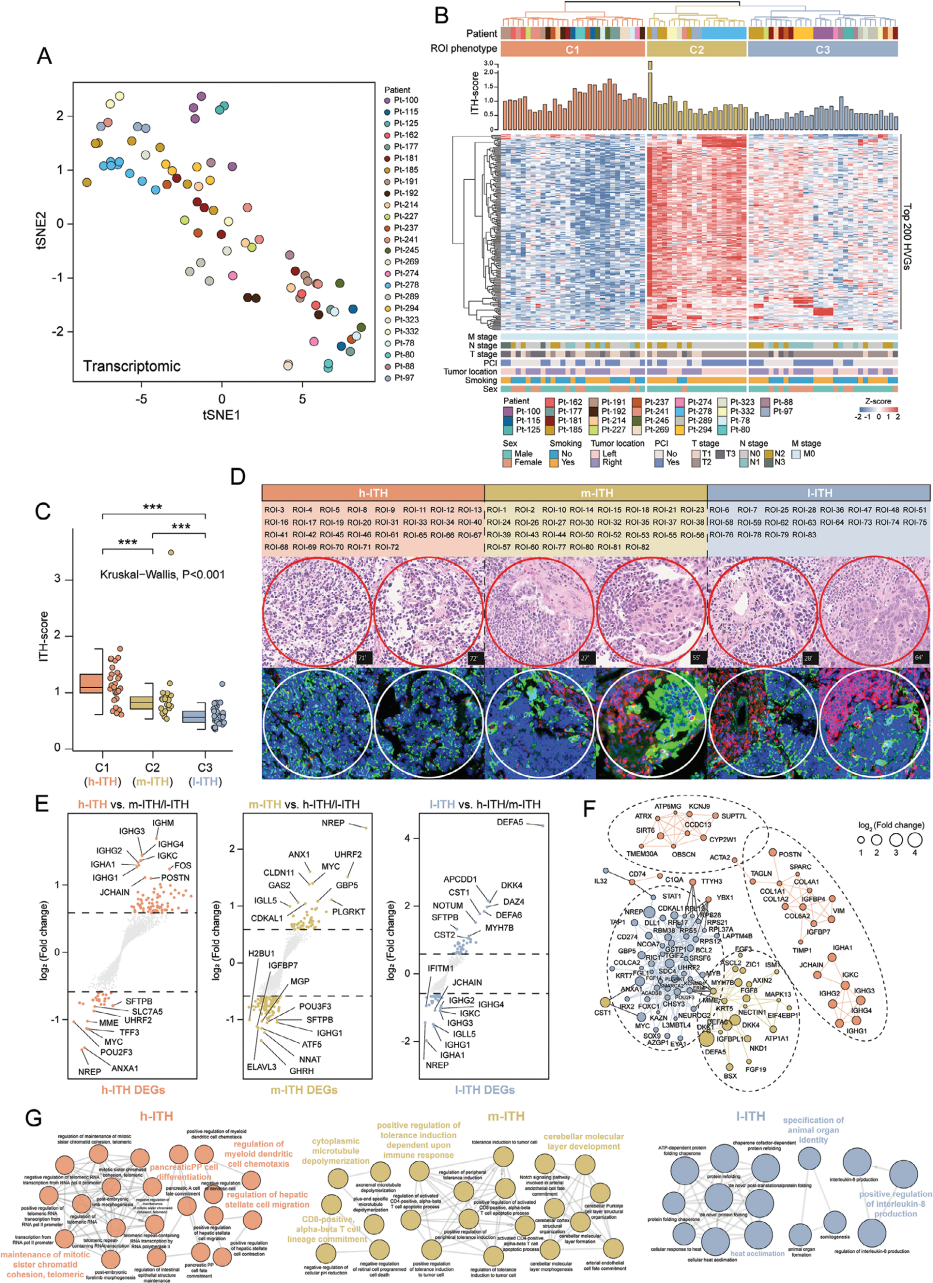
Distinct expression patterns associated with intra‐tumoral ROIs identified by DSP. A) 2D t‐SNE plot of all ROIs based on t‐SNE using the DSP transcriptomic profile. B) Heatmap showing the hierarchical clustering of 75 ROIs distributed across three distinct clusters based on the top 200 HVGs. C) Box plots showing the ITH scores among three ROI clusters. C1 is the high‐ITH phenotype (referred to as h‐ITH), C2 is the medium‐ITH phenotype (referred to as m‐ITH), and C3 is the low‐ITH phenotype (referred to as l‐ITH). *p* values were calculated with the Wilcoxon test (two clusters) and the Kruskal–Wallis test (three clusters); ns *p* > 0.05; ^*^ *p* < 0.05; ^**^ *p* < 0.01, ^***^ *p* < 0.001. D) H&E staining of the SCLC tumor tissue with ROI information. Red represents the h‐ITH ROIs; yellow represents the m‐ITH ROIs; blue represents the l‐ITH ROIs. E) Volcano plot showing differentially expressed genes among different ITH phenotypes (h‐ITH vs m‐ITH/ l‐ITH; m‐ITH vs h‐ITH/ l‐ITH and l‐ITH vs h‐ITH/ m‐ITH). The specific expressed genes (*FC *> 1.5, *p* ≤ 0.05) of each phenotype were highlighted with corresponding ITH phenotype color. F) Gene‐gene co‐expression network with color‐annotated ITH phenotype. G) Network plot showing the enriched biological process in h‐ITH phenotype, m‐ITH phenotype, and l‐ITH phenotype by ClueGO. DSP, digital spatial profiling; HVGs, highly variable genes; ITH, intra‐tumoral heterogeneity; PCA, principal component analysis; ROIs, regions of interest.

To investigate intra‐tumoral transcriptional diversity in a spatial context, the t‐SNE algorithm was used to project gene expression profiles of 79 ROIs onto a 2D space for visualization (**Figure** **2**A).  The top 200 variable genes were extracted from the top eigenvectors obtained through dimension reduction (Table S1, Supporting Information), and unsupervised hierarchical clustering was performed based on these genes across the 79 ROIs, generating three distinct clusters (C1‐C3) to represent unique transcript‐defined phenotypes (Figure 2B). We then quantified the ITH of these tumor ROIs based on gene expression profile using the DEPTH method, and observed a higher level of ITH of the C1 cluster compared to the other two groups (C2 and C3). Here, we denoted it as high‐ITH (h‐ITH). In contrast, the C3 and C2 clusters demonstrated relatively lower levels of ITH, which we termed low‐ITH (l‐ITH) and medium‐ITH (m‐ITH), respectively (Figure 2B,C; Table S2, Supporting Information). Meanwhile, there was no statistically significant difference in SPD among the three ITH phenotype groups (Kruskal–Wallis, *p *= 0.83), and no significant correlation was observed between SPD and C‐score (Spearman *R* = −0.041, *p *= 0.65) (Figure S4A; Tables S3 and S4, Supporting Information). Further examination of the pathological characteristics within these ITH‐defined groups revealed the distinct distribution of tumor‐stroma patterns and variations in the morphology of cancer cells. The h‐ITH tumors showed high purity with sporadic stromal cells and infiltrating lymphocytes and the cancer cells exhibited a more typical bare nucleus morphology with limited cytoplasmic content (Figure 2D). In contrast, the m‐ITH and l‐ITH groups showed increased fibrotic stroma and more pronounced infiltration of lymphocytes within the tumor bed, while cancer cells showed enlarged nuclei and increased cytoplasmic content (Figure 2D). These results indicate the existence of spatially defined intra‐tumoral heterogeneity at the molecular level, which is potentially associated with the pathological features of SCLC.

To further explore the underlying mechanisms driving these distinct phenotypes, we performed differential gene expression analysis pairwise between groups (h‐ITH vs others, m‐ITH vs others, and l‐ITH vs others) to identify gene signatures associated with the ITH phenotype. We identified 109 DEGs (80 up‐regulated and 29 down‐regulated), 157 DEGs (55 up‐regulated and 102 down‐regulated), and 68 DEGs (40 up‐regulated and 28 down‐regulated) for the high‐ITH, m‐ITH, and l‐ITH phenotypes, respectively (Figure 2E). Using co‐expression network analysis based on these DEG sets, we constructed critical biological networks for each ITH subtype (Figure 2F; Figure S4B, Supporting Information), generating key regulatory genes connected by multiple edges (326 edges connecting 101 genes) (Figure 2F). Within these, the h‐ITH phenotype was associated with pathways such as epithelial‐mesenchymal transition (EMT), angiogenesis, and sensitivity to DNA damage response, whereas the m‐ITH and l‐ITH groups were mainly enriched in WNT and beta‐catenin signaling, and estrogen response (Figure 2F). GO enrichment analysis revealed that the h‐ITH phenotype was associated with cell fate and differentiation, the m‐ITH phenotype was enriched in immune‐related biological processes, and the l‐ITH phenotype showed enrichment in cellular response and organ formation (Figure 2G).

### 2.3 Distinct Immune Characteristics of ITH Subtypes in the Tumor Microenvironment

**Figure 3:**
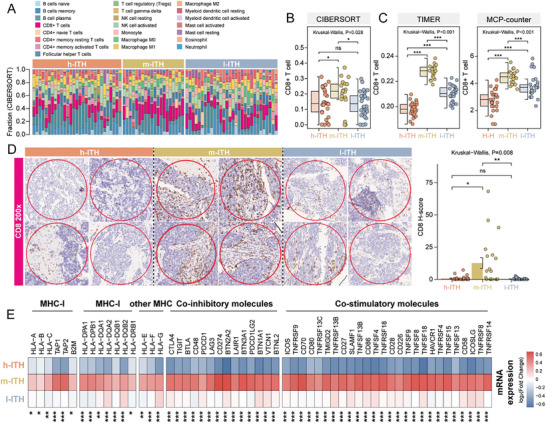
Characterization of immune features in the intra‐tumoral spatially sub‐tumor microenvironments. A) Bar plot showing the relative infiltration abundance of 22 immune cells estimated via spatial RNA expression using the CIBERSORT algorithm. B,C) Box plots showing infiltration abundance of CD8+ T cells among h‐ITH, m‐ITH, and l‐ITH phenotypes estimated by CIBERSORT, TIMER, and MCPCOUNTER algorithms. *p* values were calculated with the Wilcoxon test (two clusters) and the Kruskal‐Wallis test (three clusters); ns *p* > 0.05; ^*^ *p* < 0.05; ^**^ *p* < 0.01, ^***^ *p* < 0.001. D) IHC staining images of CD8 for h‐ITH, m‐ITH, and l‐ITH phenotypes. Bar plots showing the difference of CD8 H‐score among h‐ITH, m‐ITH and l‐ITH phenotypes. Error bars represent mean ± SEM. *p* values were calculated with the Wilcoxon test (two clusters) and the Kruskal–Wallis test (three clusters); ns *p* > 0.05; ^*^ *p* < 0.05; ^**^ *p* < 0.01, ^***^ *p* < 0.001. E) Heatmap showing expression levels of co‐stimulatory and co‐inhibitory molecules among h‐ITH, m‐ITH, and l‐ITH phenotypes. *p* values were calculated by the Wilcoxon test (two clusters) and the Kruskal‐Wallis test (three clusters); ns *p* > 0.05; ^*^ *p* < 0.05; ^**^ *p* < 0.01, ^***^ *p* < 0.001.

Given the distinct molecular features observed in ITH subtypes, particularly regarding immune‐related profiles, we focused on characterizing the spatial composition of immune cells. We conducted the deconvolution analysis from gene expression data using the CIBERSORT algorithm to quantify infiltrating immune cells at the ROI level. Among the 22 cell types quantified, four immune cell populations (CD8+ T cells, plasma B cells, CD4+ naive *T*‐cells, and resting myeloid dendritic cells) varied between ITH groups (**Figure** **3**A; Figure S5, Supporting Information). CD8+ T cells were significantly more abundant in ROIs with the m‐ITH phenotype compared to the other two phenotypes (*p *< 0.05, Figure 3B). This observation was further confirmed by two others widely used methods, TIMER and MCP‐counter (*p *< 0.05, Figure 3C). These computationally inferred findings were subsequently validated using conventional immunohistochemistry (IHC) targeting the T cell surface antigen CD8, where we observed a higher infiltration of CD8+ *T*‐cells in the ROIs of the m‐ITH phenotype compared to the other ITH phenotypes (Kruskal–Wallis, *p *= 0.008) (Figure 3D).

Since *T*‐cells mainly function via MHC‐I molecules in a dynamic interplay with various co‐regulatory cells such as dendritic cells and B cells,^[^ ^9^ ^]^ we further investigated genes involved in antigen‐presenting and surface immunoregulatory checkpoints. By averaging the expression of these genes within each ITH group, we observed distinct expression patterns associated with each ITH group. The h‐ITH or l‐ITH phenotypes showed lower expression of MHC I‐related antigen presenting molecules compared to the m‐ITH phenotype (*p *< 0.05, Figure 3E). Similarly, when comparing co‐stimulatory and co‐inhibitory molecules between ITH phenotypes, the m‐ITH phenotype showed higher expression of co‐stimulatory and immune checkpoint molecules than the other ITH phenotypes (*p *< 0.05, Figure 3E). These results indicated a sub‐histologically defined microenvironment arising from the tumor compartment with distinct ITH characteristics, potentially explained by dynamically altered molecular pathways with increased local immunogenicity involving CD8 *T*‐cell activation in the m‐ITH group.

### 2.4 Spatial Intra‐Tumoral Heterogeneity Associated with Survival and Therapeutic Outcomes

**Figure 4:**
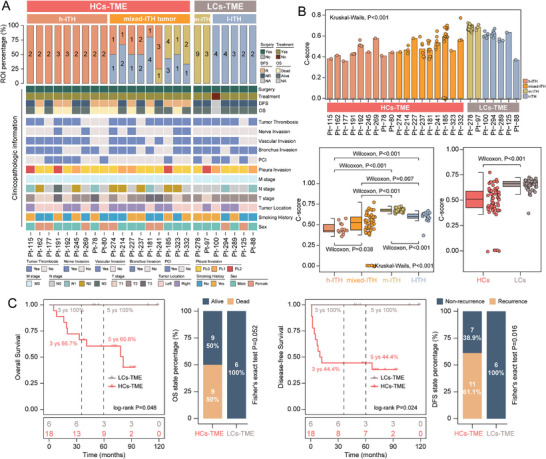
Spatial intra‐tumoral heterogeneity is associated with patient's survival and therapeutic outcome. A) The percentage of ROI ITH phenotypes at the SCLC patient's level. B) Bar plot showing the distribution of C‐score among different patient groups. *p* values were calculated with the Wilcoxon test (two clusters) and the Kruskal‐Wallis test (three clusters); ns *p* > 0.05; ^*^ *p* < 0.05; ^**^ *p* < 0.01, ^***^ *p* < 0.001. C) Kaplan–Meier analysis of OS and DFS between patients with HCs‐TME and LCs‐TME. *P* values were calculated with the log‐rank test. Stacked bar plots showing the distribution of OS and DFS status on HCs‐TME and LCs‐TME in SCLC patients. *p* values were calculated using Fisher's exact test. DFS, disease‐free survival; OS, overall survival.

To gain a deeper understanding of intra‐tumoral heterogeneity at the patient's level, we aggregated the pre‐determined ITH subtypes (low, medium, or high ITH) of individual ROIs for all patients. After mapping these ROIs back to the patients, we categorized 25 SCLC patients into two distinct TME groups: high‐complex sub‐TME (HCs‐TME) and low‐complex sub‐TME (LCs‐TME) (**Figure** **4**A). By quantifying the similarity of transcriptional features between any two ROIs within a given tumor using the C‐score, we observed a significantly higher level of transcript‐defined intra‐tumoral concordance in LCs‐TME tumors compared to HCs‐TME tumors (Kruskal–Wallis, *p *< 0.001) (Figure 4B). Moreover, these two patient groups showed significant differences in survival outcomes and therapeutic responses. LCs‐TME tumors demonstrated significantly better overall survival (OS) (log‐rank test, *p *= 0.046) and disease‐free survival (DFS) (log‐rank test, *p *= 0.024; Figure 4C) compared to HCs‐TME tumors. Notably, LCs‐TME tumors showed no death or tumor recurrence, highlighting the clinical value of this ITH classification (Figure 4C), whereas examining the clinicopathologic characteristics of the two sub‐TME phenotypes only provided limited value for patient stratification (Figure S6, Supporting Information). These findings suggest that spatial intra‐tumoral TME heterogeneity in SCLC influences patient outcomes and provides a quantitative estimate for improved patient stratification in SCLC.

### 2.5 ITH Reveals Intra‐Tumoral Molecular Subtype Heterogeneity

**Figure 5:**
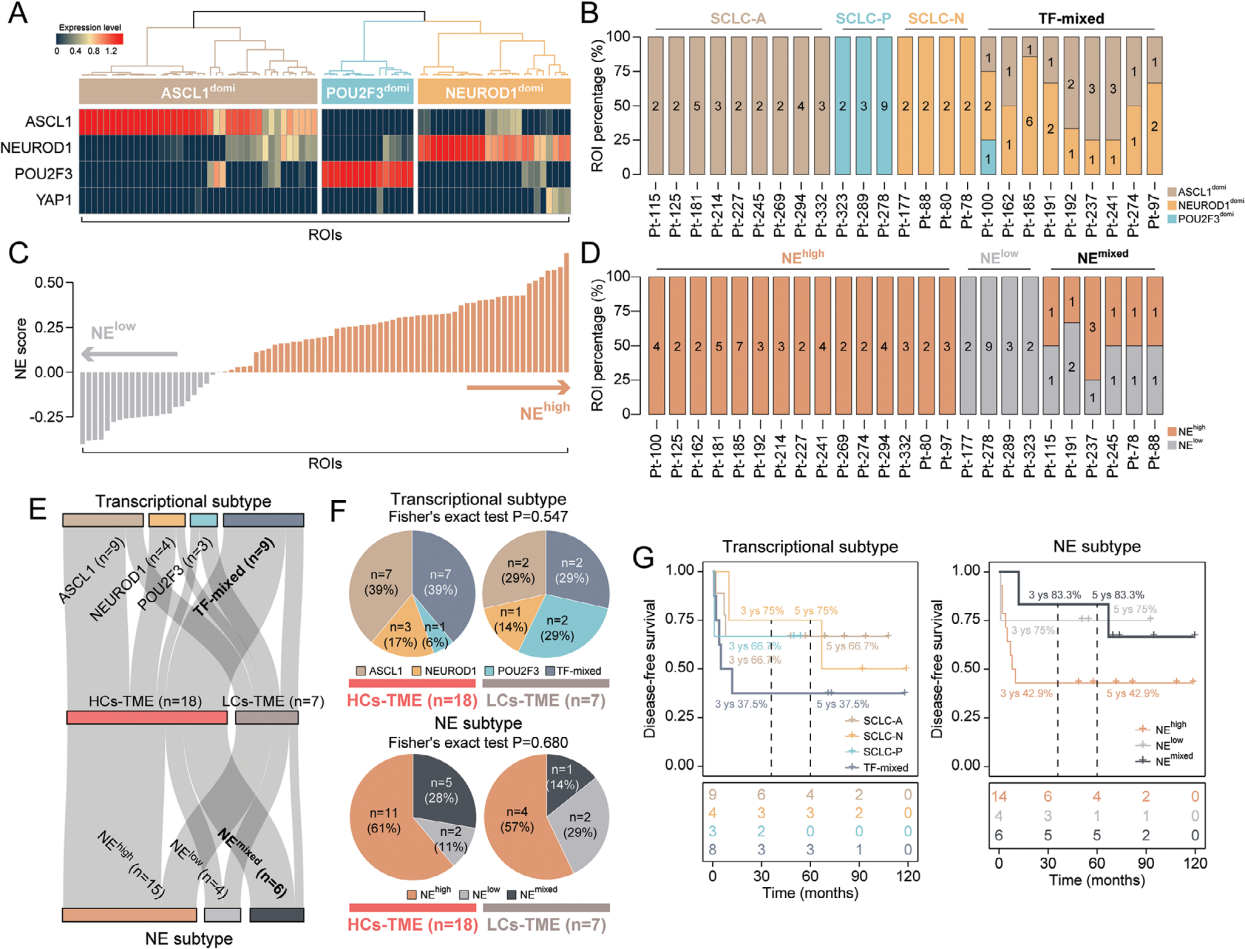
Association of spatial intra‐tumoral heterogeneity with conventional molecular classification in SCLC. A) Heatmap showing the dominant transcriptional subtype of ROIs. B) Bar plot showing the percentage of transcriptional subtypes in ROIs at the SCLC patient level. C) Bar plots showing the distribution of NE subtype of ROIs. D) Bar plot showing the percentage of ROI NE subtype at SCLC patient level. E) Sankey diagram showing the association of the sub‐TME heterogeneous phenotypes with transcriptional or NE subtypes. F) Pie plots showing the distribution of conventional molecular subtypes in HCs‐TME and LCs‐TME. *p* values were calculated using Fisher's exact test. G) Kaplan–Meier analysis of disease‐free survival among different patient groups. *p* values were calculated with the log‐rank test.

To explore the correlation between tumor spatial heterogeneity patterns and the previously established molecular subtypes (SCLC‐A, SCLC‐N, SCLC‐P, and SCLC‐Y) of SCLC defined by RNA‐seq, we extracted the spatial expression of four key transcription regulators (*ASCL1, NEUROD1, YAP1* and *POU2F3*) as previously described by Rudin et al.^[^ ^10^ ^]^ to assess SCLC molecular subtypes across 79 ROIs in 25 patients. Our analysis revealed that SCLC‐A was the most common subtype (*n* = 39, 49.4%), followed by SCLC‐N (*n* = 25, 31.6%) and SCLC‐P (*n* = 15, 19.0%) (**Figure** **5**A). We observed a high concordance at the patient level, with 59.5% of tumor ROIs from the same patient belonging to the same molecular subtype. However, nine patients had mixed ROI molecular subtypes that could not be assigned to a single molecular subtype (Figure 5B). A similar analysis was performed for neuroendocrine (NE) subtyping based on the 50 NE‐related genes, as previously described by Zhang et al.^[^ ^11^ ^]^ Consistent with the above subtype‐defined proportions, based on the NE scores calculated on each ROI, 23 ROIs were classified as low NE subtype (29.1%), and 56 ROIs were classified as high NE subtype (70.9%), confirming the above findings (Figure 5C). When comparing the NE subtyping of ROIs at the patient level, we observed that the classification of the ROI‐based NE subtyping matched the patient‐level assignment in 19 cases, but discordance occurred in six cases (Figure 5D).

Further analysis examined the concordance and discordance between intra‐tumoral sub‐TME heterogeneity‐defined SCLC subtypes and these two patient‐level molecular subtypes. No significant differences were found in the distribution of molecular subtypes (transcriptional subtypes and NE subtypes) between high TME heterogeneity (HCs‐TME) and low TME heterogeneity (LCs‐TME) patient groups (Fisher's exact test, *p *> 0.05; Figure 5E,F), suggesting that tumor‐derived heterogeneity estimation may be independent of previous transcriptional and NE‐based subtyping. Furthermore, compared to patients with a single transcriptional subtype (5‐year survival rate: 66.7% for SCLC‐A and 75% for SCLC‐N), patients with an intra‐patient mixture of different transcriptional subtypes had a worse DFS rate (5‐year survival rate: 37.5%). In contrast, patients with an intra‐patient mixture of different NE molecular subtypes or NE^low^ showed a better DFS rate (5‐year survival rate: 83.3%) compared to patients with pure NE^high^ (5‐year survival rate: 75% for NE^low^ and 42.9% for NE^high^) (Figure 5G). These results suggest that classical SCLC molecular subtypes may not be sufficiently predictive of clinical outcomes, and instead, examining intra‐patient molecular subtype diversity in a spatial context may provide more clinically relevance for patient stratification.

### 2.6 A Spatially Identified ITH‐related Molecular Signature for Prognostic Stratification

**Figure 6:**
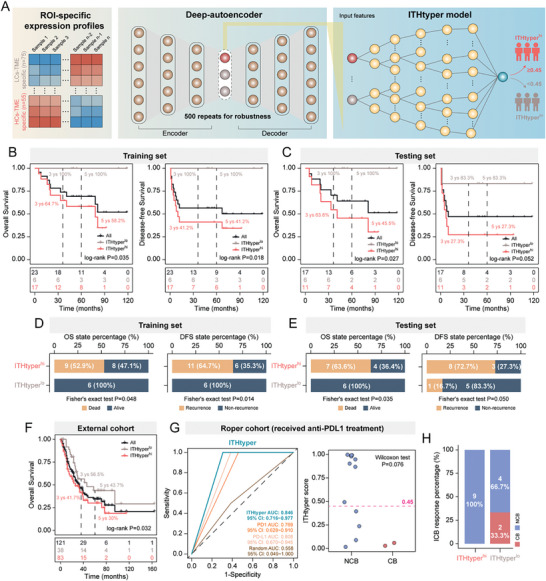
Deep learning identified spatially resolved gene signatures. A) Workflow for the computational strategy used to identify gene signature in distinguishing HCs‐TME and LCs‐TME. B,C) Kaplan–Meier analysis of OS and DFS between ITHtyper^lo^ phenotype and ITHtyper^hi^ phenotype in the training and testing sets. *P* values were calculated with the log‐rank test. D,E) Bar plots showing the distribution of OS status and DFS status on the ITHtyper^lo^ phenotype and ITHtyper^hi^ phenotype. *P* values were calculated using Fisher's exact test. F) Kaplan‐Meier analysis of OS between ITHtyper^lo^ phenotype and ITHtyper^hi^ phenotype on George & Jiang cohort (*n* = 121). *p*‐value was calculated with the log‐rank test. G) ROC curves for ITHtyper on Roper cohort (received anti‐PDL1 treatment). Dot plot showing the difference in the ITHtyper score between the NCB group and the CB group receiving immunotherapy. *p*‐value was calculated using the Wilcoxon test. H) Bar plot showing the distribution of ICB response on ITHtyper^lo^ phenotype and ITHtyper^hi^ phenotype. DFS, disease‐free survival; OS, overall survival; ROC, Receiver operating characteristic; CB, clinical benefit; NCB, no clinical benefit.

To investigate the potential of ITH region‐specific gene expression to infer tumor‐originating TME phenotypes, we used a deep‐autoencoder framework to select features based on the DSP data that could effectively discriminate HCs‐TME from LCs‐TME. Ten genes (*NKX1‐2*, *TLE2*, *TPBG*, *GPR31*, *SRSF6*, *DAZ4*, *PD‐L1*, *LYZ*, *PCP4* and *ZIC1*) were identified based on their significance values, and then used to develop a machine‐learning model (named ITHtyper) based on the XGBoost algorithm for sub‐TME heterogeneity subtyping (**Figure** **6**A). The ITHtyper was trained on 70% of the patients and evaluated on the remaining 30% for internal validation. The ITHtyper effectively dichotomized SCLC ROIs into high ITHtyper (ITHtyper^hi^) and low ITHtyper (ITHtyper^lo^) phenotypes using a threshold of 0.45, corresponding to HCs‐TME and LCs‐TME, respectively. Survival analyses on both the training and testing sets demonstrated that the ITHtyper^lo^ phenotype was associated with improved OS and DFS (log‐rank test, *p *= 0.035 and 0.027 for OS, and *p *= 0.018 and 0.052 for DFS, respectively) (Figure 6B,C). Notably, the ITHtyper^lo^ phenotype showed no death or tumor recurrence (Figure 6D,E). To further validate the applicability of ITHtyper in bulk RNA‐seq profiles, we applied it to 121 bulk tissue RNA‐seq profiles from multicenter SCLC patient cohorts. The results showed that patients (*n* = 38) with the ITHtyper^lo^ phenotype had significantly better survival outcomes compared to those (*n* = 83) with the ITHtyper^hi^ phenotype (log‐rank test, *p *= 0.032; Figure 6F), highlighting its predictive potential for prognostic stratification. Additionally, when applied to immunotherapy cohorts, the ITHtyper showed superior predictive power for immunotherapy response (AUC = 0.846) compared to PD1 (AUC = 0.769) and PD‐L1 (AUC = 0.808) (Figure 6G). Notably, all patients who achieved complete response to immunotherapy belonged to the ITHtyper^lo^ phenotype group, implying its broader potential as a biomarker for predicting ICI response (Figure 6H).

## 3 Discussion

SCLC has historically been considered a homogeneous malignancy, genetically characterized by universal loss of the TP53 and RB1 genes.^[^ ^12^ ^]^ However, recent technological advances have uncovered a previously unrecognized complexity and transformed our understanding of the intra‐tumoral heterogeneity of SCLC.^[^ ^13^ ^]^ Accumulating evidence from both molecular and clinical studies has led us to recognize SCLC as a diverse entity, encompassing a spectrum of molecular subtypes within individual tumors and even between different tumors.^[^ ^7^, ^8^, ^13^, ^14^ ^]^ Elucidating the inherent heterogeneity is critical to understand the mechanistic drivers of its aggressiveness and to facilitate the development of novel therapeutic strategies. Currently, most studies of ITH in SCLC have relied on mouse models, circulating tumor cells (CTCs), or samples from chemotherapy‐resistant patients with advanced disease, using either bulk transcriptomics or single‐cell approaches.^[^ ^7^, ^14^ ^]^ However, a critical knowledge gap remains regarding the extent of heterogeneity revealed by in‐depth molecular profiling at the spatial level, particularly in treatment‐naïve SCLC patients in a clinical setting.

Recent advances in spatial transcriptomics, such as 10xVisium or Stereo‐seq, have enabled high‐resolution mapping of gene expression in intact cell and tissue samples.^[^ ^15^ ^]^ In this study, we aimed to address tumor‐enriched spatial transcriptomic profiles rather than a complete spatial architecture. From a clinicopathological perspective, we hypothesize that the histological structure of subregional ecosystems is intricately linked to the biological behavior of tumors. To investigate this, we conducted a comparative study using different subregions of clinically archived pathological specimens and correlated them with patient outcomes. Our ROI‐based approach provided an intra‐tumoral picture, allowing in‐depth exploration of molecular patterns, biological pathways, and immunological features central to ITH in SCLC from a multi‐regional perspective. To ensure the robustness of our analysis, we controlled for sampling bias in two main ways. First, we rigorously excluded cases of combined SCLC (notorious for its heterogeneity with a mixture of different non‐small cell components). In addition, we excluded multi‐regional sampling bias by comparing SPD between any ROIs. Based on 25 SCLC patients, we identified a spatially resolved gene signature delineating three distinct ITH characteristics in SCLC. These results highlight the presence of discrete tumor clones, each with unique molecular features, even within morphologically identical regions, consistent with the concept of molecular heterogeneity introduced in a previous study.^[^ ^14c^ ^]^ Our ITH‐based classification not only revealed the canonical neuroendocrine characteristics of SCLC in those with intermediate to low ITH, but also implied a highly active EMT transition in ITH‐high tumors with increased metastatic potential.^[^ ^16^ ^]^

Our study also delved into the realm of immune cell composition, and identified CD8+ *T*‐cells as a critical indicator associated with ITH status, which was confirmed by conventional IHC. Specifically, tumors with intermediate ITH exhibited a “hot” immune microenvironment, enriched for CD8+ *T*‐cell infiltration and increased expression of antigen‐presenting molecules, co‐stimulatory molecules, and immune checkpoints. In contrast, tumors with either high or low ITH exhibited lower levels of local immunogenicity. These findings, consistent with previous studies,^[^ ^17^ ^]^ suggest crosstalk between tumor cells and TIME. These tumor‐originated dynamics may dictate regional immune heterogeneity, with the reactive subtype (m‐ITH) exhibiting an inflamed TIME consistent with Tian's findings of the interplay between cancer cell ITH and the TME at the single‐cell level.^[^ ^7b^ ^]^ Furthermore, ITH‐associated meta‐programs in malignant tumor cells showed a negative correlation with CD8+ *T*‐cells cytotoxicity and cell cycle meta‐programs,^[^ ^18^ ^]^ suggesting a resistant microenvironment within highly heterogeneous tumors that impedes the recruitment of cytotoxic *T*‐cells. Crucially, the spatial mapping of all ROIs at the patient level highlights the significant impact of ITH on patient survival and therapeutic outcomes. These findings suggest a potential role for high ITH in immune suppression and/or evasion, ultimately facilitating metastatic potential and leading to poorer patient prognosis. In light of recent data demonstrating the benefit of immune checkpoint inhibitors in combination with chemotherapy for a subset of patients with extensive‐stage SCLC,^[^ ^2^ ^]^ our findings hold promise to aid in the stratification of SCLC patients using ITH‐associated transcriptional profiles.

Another interesting finding of our study is the presence of ITH across molecular subtypes, highlighting the existence of different molecular subtypes within different spatial regions of the same tumor. These observations shed light on the challenges of current molecular subtyping methods, including issues with prognostic stratification and low reproducibility.^[^ ^8^ ^]^ Furthermore, these findings indicate that SCLC molecular subtypes do not manifest uniformly across spatially distinct regions within the same tumor. Instead, the intra‐individual diversity of molecular subtypes is distributed across different tumor regions. This molecular heterogeneity may be due to the neuroendocrine nature of SCLC itself, which may explain the challenges in applying current transcriptional subtyping approaches for clinical purposes.

The ITHtyper, developed through our ITH subtyping, is a valuable tool for distinguishing the clinical phenotypes of SCLC. This strategy is consistent with similar efforts to identify gene sets associated with heterogeneity in other malignancies, such as glioblastoma and melanoma, where specific genes have been linked to key drivers of tumor heterogeneity.^[^ ^19^ ^]^ Our current study used a deep‐autoencoder‐based algorithm and a bootstrap strategy to identify the top 10 significant genes associated with SCLC heterogeneity. These genes include those involved in EMT, transcriptional regulation, growth and development, and the immune checkpoint inhibitor *PD‐L1*. For example, the *TPBG* and *GPR31* genes are highly correlated with the HCs‐TME subset and have known associations with tumor cell migration, proliferation, and recurrence via EMT.^[^ ^20^ ^]^ *PCP4*, specific for the LCs‐TME subset, is a potential therapeutic target as it inhibits EMT and promotes apoptotic cell death.^[^ ^21^ ^]^ In addition, Z*IC1*, which is frequently inactivated by promoter hypermethylation, acts as a tumor suppressor by modulating PI3K/Akt and MAPK pathways and influencing EMT in several cancers, including thyroid, breast, and gastric cancers.^[^ ^22^ ^]^

However, it is important to acknowledge the limitations of our study.  First, our experimental cohort focuses primarily on treatment‐naïve limited‐stage tumors, excluding extensive‐stage specimens and those treated with chemotherapy or chemoimmunotherapy before surgery. However, we validate our critical findings in independent cohorts, thereby strengthening the generalizability of our results beyond limited‐stage SCLC cases. Second, while our study efficiently identifies significant alterations in transcriptional expression, other omics data, such as the protein or metabolite level, can be explored to cross‐validate our findings. Subsequent investigations integrating spatial proteomic and metabolomic assessments will provide a more comprehensive understanding of ITH in SCLC. Third, the spatial sampling of ROIs was performed at a “small‐bulk” level, potentially leading to an underestimation of subclonal diversity at the single‐cell level. The use of single‐cell resolution in situ has the potential to improve our understanding of the heterogeneity present in SCLC tumors. However, the definition and transcriptional profiling of sub‐regional ITH in our study is more consistent with our previous pathological observations and prone to be clinicallymeaningful, which was performed under thorough pathological guidance beyond the single‐cell level. Furthermore, it is crucial to consider the spatial and temporal heterogeneity in the context of tumor evolution. An analysis of the dynamic alterations in ITH over time, coupled with longitudinal sampling, is essential for a comprehensive understanding of tumor evolution and its impact on treatment response.

## 4 Conclusion

In conclusion, this study provided a spatial multi‐regional transcriptomic landscape of SCLCs and uncovered unique spatial transcriptomic features characterized by distinct molecular profiles, underlying biological processes, immune phenotypes, and molecular subtype diversity. These features correlate significantly with patient prognosis and shed light on intra‐tumoral heterogeneity. This spatially subregion‐focused investigation provides an invaluable resource for studying tumorigenic ITH and elucidates its underlying mechanisms and interplay with the TIME. Leveraging machine learning to classify tumors based on ITH scores represents an initial step towards understanding SCLC in a spatially resolved framework, with the potential to pave the way for improved patient stratification in the clinical translational setting.

## 5 Experimental Section

### Study Cohorts

The study cohort consisted of 25 treatment‐naïve patients with limited‐stage SCLC (LS‐SCLC) patients who underwent clinical resection. These patients were recruited from the archived electronic medical record system (EMRS) at the Department of Pathology, Cancer Hospital, Chinese Academy of Medical Sciences (CHCAMS) between 2009 and 2016, and all were pathologically confirmed. Detailed clinical and pathological information of this cohort is shown in **Table** **1**. The inclusion criteria were as follows: 1) no preoperative treatment; 2) availability of complete clinical and follow‐up data, including disease‐free survival (DFS) and overall survival (OS); 3) pathological confirmation of pure SCLC by routine differential immunostaining for neuroendocrine biomarkers (ChrA, CD56, Syno) and TTF‐1, Ki‐67, according to the 5th World Health Organization thoracic tumor classification.^[^ ^1a^ ^]^ This retrospective study was approved by the Ethics Committee and Institutional Review Boards of the National GCP Center (approval number 22/250‐3452), and all patients were exempted from informed consent due to the retrospective nature of this study.

**Table 1:** Clinicopathological characteristics of SCLC patients.

Characteristics	CHCAMS cohort [*n* = 25]	Jiang cohort [*n* = 50]	George cohort [*n* = 77]	Roper cohort [Immunotherapy, *n* = 16]
Sample type	SCLC	SCLC	SCLC	SCLC
Sex [n, (%)]				
Male	16 (64%)	45 (90%)	52 (67.5%)	8 (50%)
Female	9 (36%)	5 (10%)	25 (32.5%)	8 (50%)
Age [n, (%)]				
< = 65	22 (88%)	38 (76%)	41 (53.2%)	11 (68.75%)
>65	3 (12%)	12 (24%)	36 (46.8%)	5 (21.25%)
Smoking [n, (%)]				
Yes	12 (48%)	35 (70%)	75 (97.4%)	14 (87.5%)
No	13 (52%)	15 (30%)	2 (2.6%)	2 (12.5%)
Unknown	0	0	0	0
Tumor location [n, (%)]				
Left	12 (48%)	0	0	0
Right	13 (52%)	0	0	0
Unknown	0	50 (100%)	77 (100%)	16 (100%)
PCI [n, (%)]				
Yes	10 (40%)	0	0	0
No	15 (60%)	0	0	0
Unknown	0	50 (100%)	77 (100%)	16 (100%)
DFS status [n, (%)]				
Recurrence	11 (44%)	0	0	0
Non‐recurrence	13 (52%)	0	0	0
Unknown	1 (4%)	50 (100%)	77 (100%)	16 (100%)
OS status [n, (%)]				
Alive	15 (60%)	23 (46%)	29 (37.7%)	1 (6.25%)
Dead	9 (36%)	25 (50%)	44 (57.1%)	15 (93.75%)
Unknown	1 (4%)	2 (4%)	4 (5.2%)	0
T stage [n, (%)]				
T1	7 (28%)	9 (18%)	28 (36.4%)	0
T2	14 (56%)	28 (56%)	28 (36.4%)	0
T3	4 (16%)	8 (16%)	6 (7.8%)	0
T4	0	4 (8%)	5 (6.5%)	0
Unknown	0	1 (2%)	10 (13.0%)	16 (100%)
N stage [n, (%)]				
N0	12 (48%)	11 (22%)	33 (42.9%)	0
N1	5 (20%)	5 (10%)	11 (14.3%)	0
N2	7 (28%)	32 (64%)	21 (27.3%)	0
N3	1 (4%)	1 (2%)	2 (2.6%)	0
Unknown	0	1 (2%)	10 (13.0%)	16 (100%)
M stage [n, (%)]				
M0	25 (100%)	48 (96%)	54 (70.1%)	0
M1	0	1 (2%)	8 (10.4%)	0
Unknown	0	1 (2%)	15 (19.5%)	16 (100%)
Response [n, (%)]				
CB	0	0	0	2 (12.5%)
NCB	0	0	0	13 81.25%)
Unknown	25 (100%)	50 (100%)	77 (100%)	1 (6.25%)

### Tissue Microarray (TMA) Construction

A customized TMA was prepared by two experienced pathologists who thoroughly reviewed sections from all patients to identify representative tumor areas. Two representative cores, each with a diameter of 1.5 mm, were extracted from the tumor central region of each patient and placed on a recipient TMA master block. Areas of necrosis, fibrosis, hemorrhage, and cystic changes were excluded during core selection. Following TMA preparation, serial sections were prepared for pathologic staining and spatial profiling, respectively. One section was stained with hematoxylin and eosin (H&E) for pathological characterization of more than 90% neoplastic content to ensure tumor purity. The parallel sections were then used for spatial transcriptome profiling.

### ROI Selection Strategy for Spatial Profiling

To prepare for spatial profiling, 4 µm thick TMA sections were deparaffinized, antigen retrieved, and simultaneously stained with target‐detecting probes and concurrent morphologic antibodies (details below). To visualize the TME, fluorescent antibodies specific for the pan‐leukocyte marker CD45 (CST 13917), the epithelial cell marker PanCK (Novus NEP2‐33200), and the nuclear stain SYTO13 (NanoString, 121300310) were used for morphological staining. After staining and hybridization, the slides were loaded onto the GeoMx Digital Spatial Profiler (DSP) instrument (NanoString, Seattle, USA) and scanned to generate fluorescent tissue images before ROI selection. Tri‐color immunofluorescence was used to identify tumor cells, infiltrating lymphocytes, and all nucleated cells in the tumor‐enriched area (excluding necrotic regions). ROIs, each with a 600 µm diameter and mainly in a circular shape, were then selected. After selection, each ROI was annotated by pathologists to confirm the location and content of the tumor. Due to limited areas available for spatial profiling, 2–9 ROIs were selected per patient. A total of 79 ROIs were selected from 25 FFPE specimens. Near‐identical ROIs were chosen to ensure data consistency arising from highly heterogeneous tissue compartments, even at the intra‐tumoral level. The collected ROIs were then processed according to the protocols described in the following sections. The *x‐y* coordinates of each ROI, generated from the DSP data, were used for the subsequent ITH analysis.

### Transcriptome‐Wide Spatial RNA Profiling

Transcriptome‐wide spatial RNA profiling was performed using the Human Whole Transcriptome Atlas (WTA) panel developed for the DSP technology. TMA slides, prepared as described above, were hybridized with detection probes at 37 °C overnight and subjected to morphological staining. ROIs were harvested using an ultraviolet (UV)‐guided built‐in technique that allowed the deposition of photocleavable oligonucleotides into individual wells on a 96‐well plate.^[^ ^23^ ^]^ Samples were then transferred to a new plate for probe hybridization and PCR amplification. The resulting products were purified and quality assessed before being subjected to next‐generation sequencing (NGS) on an Illumina NovaSeq 6000 instrument. After sequencing, the FASTQ files were converted into digital count conversion (DCC) files, and uploaded to the DSP system to generate a count matrix for individual ROIs. For inter‐ROI comparison, the RNA data was normalized to Q3. The limit of quantitation (LOQ) for each ROI was determined using the formula: LOQ = geometric mean (NegProbe_i) × geometric standard deviation (NegProbe_i) ×10^2^ The RNA data was covered to count level and subjected to log2(x+1) normalization in this study.

### Publicly Bulk RNA‐Seq Cohorts

Three SCLC cohorts (*n* = 143) with available RNA‐seq data and clinicopathologic information from the Gene Expression Omnibus (GEO) databases and related publications were collected, including 50 patients from GSE60052 (https://www.ncbi.nlm.nih.gov/geo/query/acc.cgi?acc = GSE60052),^[^ ^24^ ^]^ 77 patients from George's study (referred to as George cohort)^[^ ^25^ ^]^ and 16 patients who received anti‐PD‐L1 antibody durvalumab in combination with olaparib from Roper's study (referred to as Roper cohort).^[^ ^26^ ^]^ The mRNA expression profile in GSE60052 was transformed to the count level and normalized to log2(x+1). The RNA‐seq data of the George cohort was normalized to the fragments per kilobase million (FPKM), and the transcriptome profile of the Roper cohort was normalized to reads per kilobase million (RPKM).

### Quantitative Assessment of Intra‐Tumoral Spatial Heterogeneity

Deviating gene Expression Profiling Tumor Heterogeneity (DEPTH), a bulk sequencing‐based approach to evaluate the ITH levels in mRNA data,^[^ ^27^ ^]^ was used to quantify intraregional ITH for each ROI based on alterations of gene expression profiles. A low DEPTH score represents low intra‐regional ITH, while a high score suggests the opposite. Spatial inter‐ROI ITH within the same tumor was quantified using the consistent score (C‐score) by calculating Spearman correlation coefficients of the transcriptomic profiles between the two ROIs being compared.^[^ ^28^ ^]^

### Gene‐Gene Functional Association Network

Differential expression analysis was performed using the R package “limma” (v3.50.3) on the Q3 normalized DSP data. Differentially expressed genes (DEGs) were identified using a threshold of *p*‐value < 0.05 and fold‐change (FC) > 1.5. The Spearman correlation coefficient was calculated to measure the co‐expression relationship between two sets of DEGs. The co‐expressed DEG pairs with Spearman correlation coefficient ≥ 0.6 and *p*‐value < 0.01 were used to construct the gene–gene functional association network. The network was built and visualized using the R package “igraph” (1.4.3).

### Functional Enrichment Analysis

Gene set enrichment analysis (GSEA) for hallmark gene sets from the Molecular Signatures Database (MSigDB, v7.2) was performed using the “enricher” function of the clusterProfiler package (v4.8.1).^[^ ^29^ ^]^ Functional enrichment analysis of Gene Ontology (GO) for specific gene sets was performed using the Cytoscape plug‐in “ClueGO” (v2.5.9).^[^ ^30^ ^]^

### Computationally Estimation of Immune Cell Infiltration

The relative abundance of 22 immune cell populations was estimated from the RNA expression data using the CIBERSORT method.^[^ ^31^ ^]^ The absolute abundance of eight immune cell populations from the transcriptomic data was quantified using the Microenvironment Cell Populations‐counter (MCP‐counter) method.^[^ ^32^ ^]^ Another complementary algorithm, Tumor Immune Estimation Resource (TIMER),^[^ ^33^ ^]^ was also used to infer the abundance of six tumor‐infiltrating immune cells based on the gene expression profiles.

### Immunohistochemistry (IHC) Staining

IHC was performed on 4 µm thick full sections obtained from selected blocks using fully automated Roche's immunohistochemical instruments (BenchMark ULTRA IHC/ISH System from Roche Diagnostics). Standard protocols were followed for the IHC procedure. After deparaffinization, sections were subjected to antigen retrieval at 97 °C for 30 min. The sections were then blocked with H_2_O_2_ for 5 min at room temperature and incubated overnight at 4 °C with primary antibodies against CD*3* (MXB, rat# abMX036) and *CD8* (ZXGB‐bio, rabbit# abSP16). After primary antibody incubation, sections were incubated with HRP‐conjugated secondary antibody for 30 min at 37 °C and developed with DAB substrate for colorimetric visualization in an autostainer (Autostainer Link 48, Dako, Denmark). Slides were scanned on the KFBIO Digital Slice Scanning System (KF‐PRO‐400‐HI) and analyzed using QuPath software v0.3.2, an open‐source and user‐friendly software used for digital evaluation and analysis of pathologic features on whole‐slide images.^[^ ^34^ ^]^ The pathologic evaluation included the calculation of a composite score that considered both the staining intensity and the percentage of positive cells, all observed at 200×magnification.

### Development of Gene Signature

The DSP data were used to develop prediction models, and the ROIs were randomly divided into a training set (70%) and a testing set (30%). A deep‐learning algorithm called “Deep‐autoencoder” was used to identify a spatially resolved gene signature. To ensure the robustness and reliability of the gene signature, the bootstrap approach was used by randomly sampling 90% of the samples (without replacement) with 500 iterations. The tuned parameters of “Deep‐autoencoder” were set as follows: activation = “Tanh”, hidden = 100, 300, and 100, and epoch = 10. An extreme gradient boosting model (XGBoost) was used to build the classifier for heterogeneity subtyping and risk stratification using the spatially resolved gene signature from above. The XGBoost was implemented in the R package “XGBoost” (v1.5.2.1). The hyperparameters for tuning were set as follows: objective = “binary: logistic”, n‐round = 100, n‐fold = 5, max depth = 5, subsample = 80%, colsample_bytree = 80%, and evaluation metrics = “error”.

### Statistical Analysis

All statistical tests and graphical visualizations were performed using the R software (v4.2.2) and the corresponding R packages within the Rstudio (v2022.12.0+353). Unless otherwise stated, Wilcoxon rank sum and Kruskal–Wallis tests were used to compare continuous variables between groups, and Fisher's exact test was used to analyze contingency table variables. High‐dimensional data were reduced to low dimensions and visualized using the t‐distributed stochastic neighbor embedding (t‐SNE) algorithm. The correlation between two continuous variables was calculated using Spearman's correlation test. Unsupervised hierarchical clustering was performed using the “ward. D2” function from the embedded R package “stats” (v4.2.2). For survival analysis, Kaplan–Meier (K‐M) plots were used to generate survival curves, and log‐rank tests were conducted to compare survival differences between patient groups using the R packages “survival” (v3.4‐0) and “survminer” (v0.4.9). Receiver operating characteristic (ROC) curves and area under the curve (AUC) were used to evaluate the performance and generalizability of the classification model. Two‐sided *p* values were considered statistically significant with a threshold of 0.05.

### Ethics Statement

This retrospective study was approved by the Ethics Committee and Institutional Review Boards of the National GCP Center (approval number 22/250‐3452), and all patients were exempted from informed consent due to the retrospective nature of this study.

## Conflict of Interest

The authors declare no conflict of interest.

## Author Contributions

M.Z., and L.Y. conceived the study. Z.C.Z., Y.B.Z., and Z.J.Y. performed the bioinformatics analysis. Y.T.L. contributed to fund acquisititon, X.J.S. performed IHC experiments J.Y.D. contributed to sample collection. Z.C.Z., M.Z., Y.T.L., X.J.S., N.W., J.M.Y., and L.Y. wrote and revised the manuscript. All authors read and approved the final manuscript.

## Supporting information

Supporting Information

Supporting Information

Supporting Information

Supporting Information

Supporting Information

## Data Availability

The raw data of spatial transcriptomics generated in this study were deposited in the Sequence Read Archive under accession number PRJNA1014231 (https://www.ncbi.nlm.nih.gov/sra). Other public SCLC cohorts with transcriptomic data and clinical information are available from the Gene Expression Omnibus (GEO) database under accession number GSE60052, as well as corresponding publications, including George's and Roper's studies. The source code of this work can be downloaded from https://github.com/ZhoulabCPH/ITHtyper.
