# Supplementary material for: Spatial Transcriptome‐Wide Profiling of Small Cell Lung Cancer Reveals Intra‐Tumoral Molecular and Subtype Heterogeneity
Source: Adv Sci (Weinh). 2024 Jun 19;11(31):2402716. doi: 10.1002/advs.202402716 (PMC11336901; doi:10.1002/advs.202402716)
Supplement: Supplementary file 1 — Supporting Information [file ADVS-11-2402716-s003.docx]

**Supplementary Information**

**Spatial Transcriptome-wide Profiling of Small Cell Lung Cancer Reveals Intra-tumoral Molecular and Subtype Heterogeneity**

Zicheng Zhang^1,2^, Xujie Sun^2^, Yutao Liu^3*^, Yibo Zhang^1^, Zijian Yang^1^, Jiyan Dong^2^, Nan Wang^4^, Jianming Ying^2^, Meng Zhou^1*^, Lin Yang^2*^

*** Correspondence should be addressed to**

Meng Zhou, zhoumeng@wmu.edu.cn (ORCID: 0000-0001-9987-9024)

School of Biomedical Engineering, National Clinical Research Center for Ocular Diseases, Eye Hospital, Wenzhou Medical University, Wenzhou 325027, P. R. China Lin Yang, yanglin@cicams.ac.cn (ORCID: 0000-0002-7594-3770)

Department of Pathology, National Cancer Center/National Clinical Research Center for Cancer/Cancer Hospital, Chinese Academy of Medical Sciences and Peking Union Medical College, Beijing 100021, P. R. China

Yutao Liu, liuyutao@cicams.ac.cn

Department of Medical Oncology, National Cancer Center/National Clinical Research Center for Cancer/Cancer Hospital, Chinese Academy of Medical Sciences and Peking Union Medical College, Beijing 100021, P. R. China.


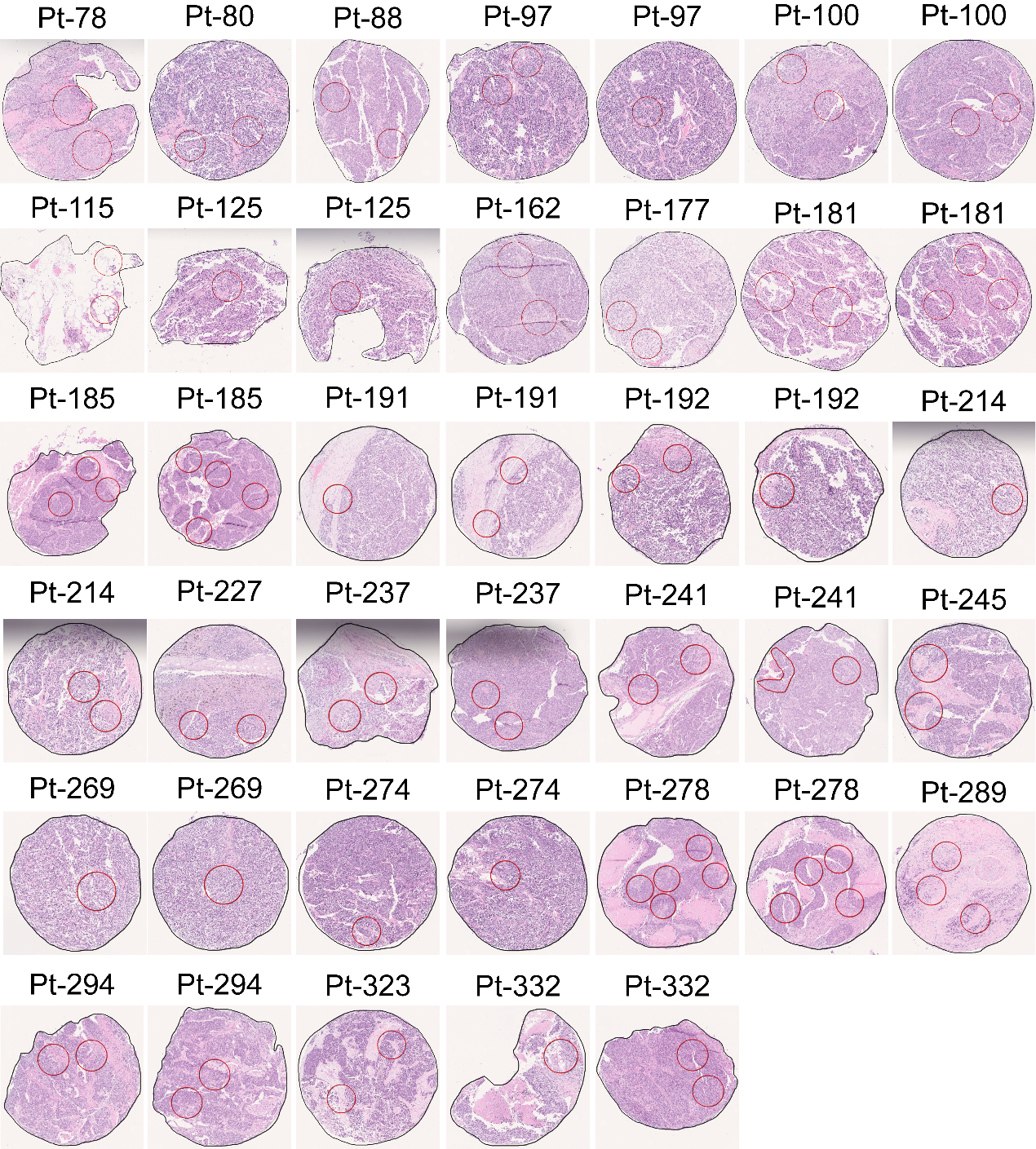


**Supplementary Figure 1. H&E images of 79 ROIs on 25 SCLC patients.**

**
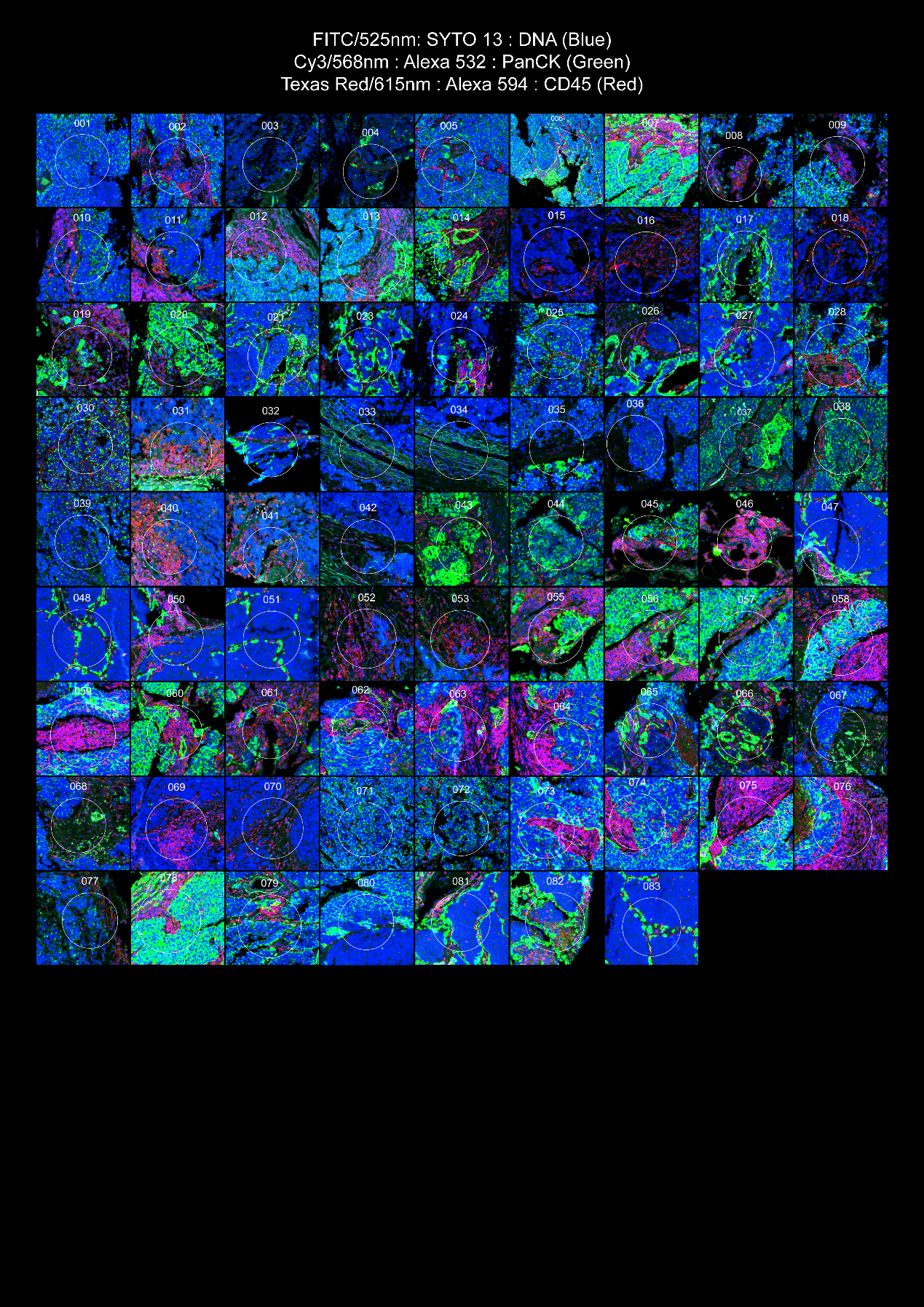
**

**Supplementary Figure 2. Fluorescent staining images of 79 ROIs on 25 SCLC patients.**

**
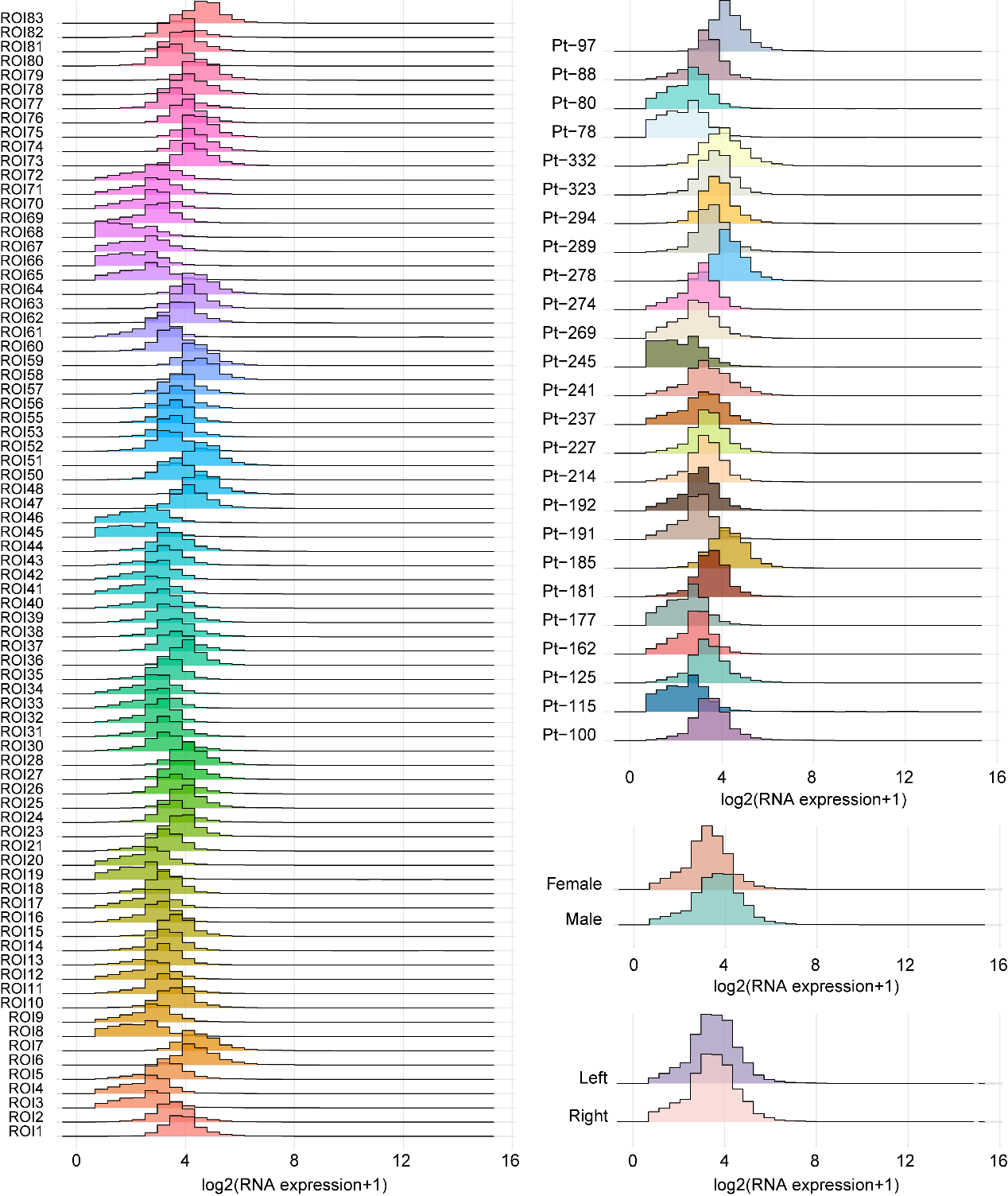
**

**Supplementary Figure 3. The distribution of RNA expression among ROIs, patients, sex, and tumor location, respectively.** The expression was transformed by log_2_(x + 1).


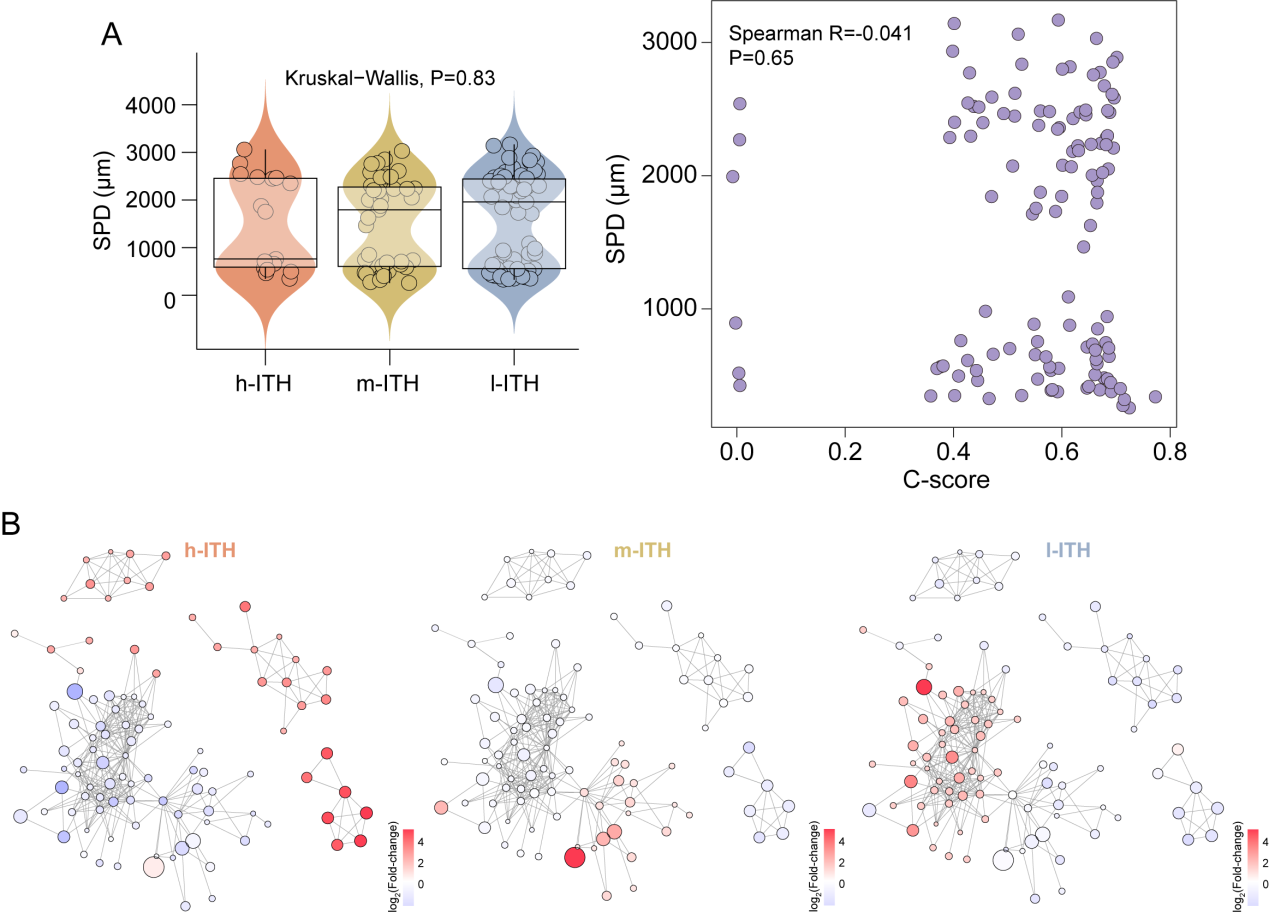


**Supplementary Figure 4. DSP analysis of 79 ROIs on SCLC patients.** (A) Box plot showing the distribution among h-ITH, m-ITH, and l-ITH phenotypes. *P* value was calculated with the Kruskal-Wallis test (three clusters). Dot plot showing the association between SPD and C-score. The *P* value was calculated using Spearman’s correlation method. (B) Network plot showing the FC of DEGs across h-ITH phenotype, m-ITH phenotype, and l-ITH phenotype.


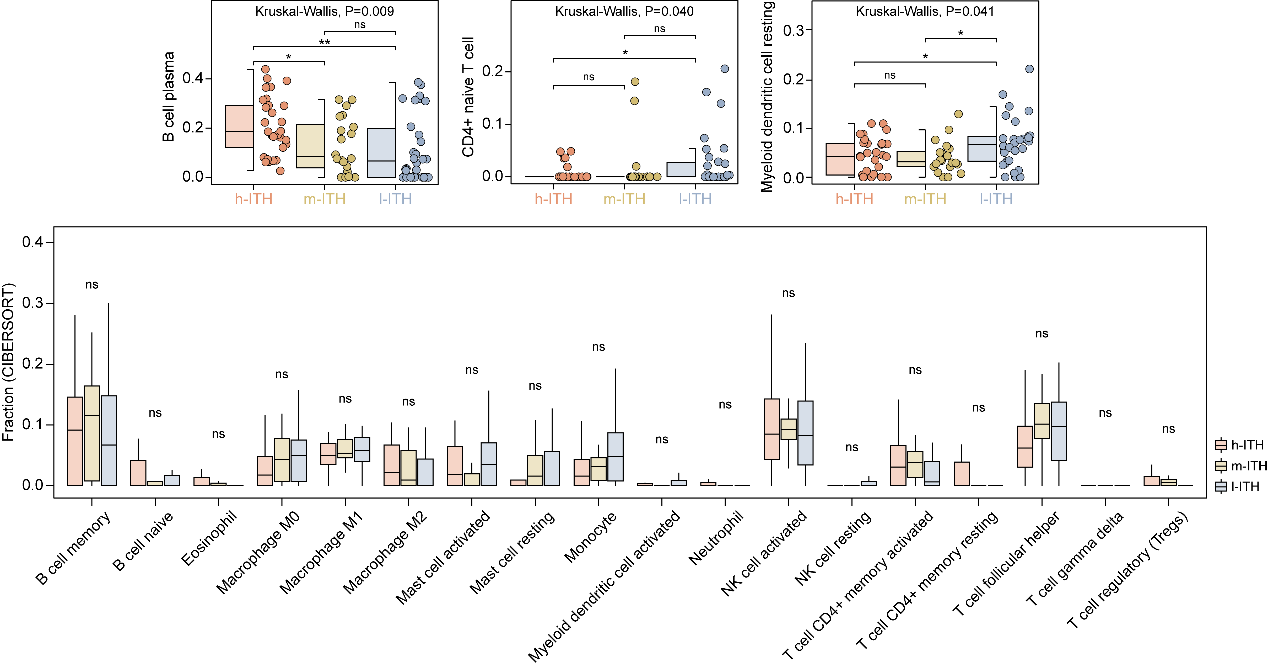


**Supplementary Figure 5. Characterization of immune cell types.** Box plots showing the distribution of immune cell infiltration among h-ITH, m-ITH, and l-ITH phenotypes. *P* values were calculated with the Wilcoxon test (two clusters) and the Kruskal-Wallis test (three clusters); ns *P* > 0.05; **P* < 0.05; ***P* < 0.01, ****P* < 0.001.

**
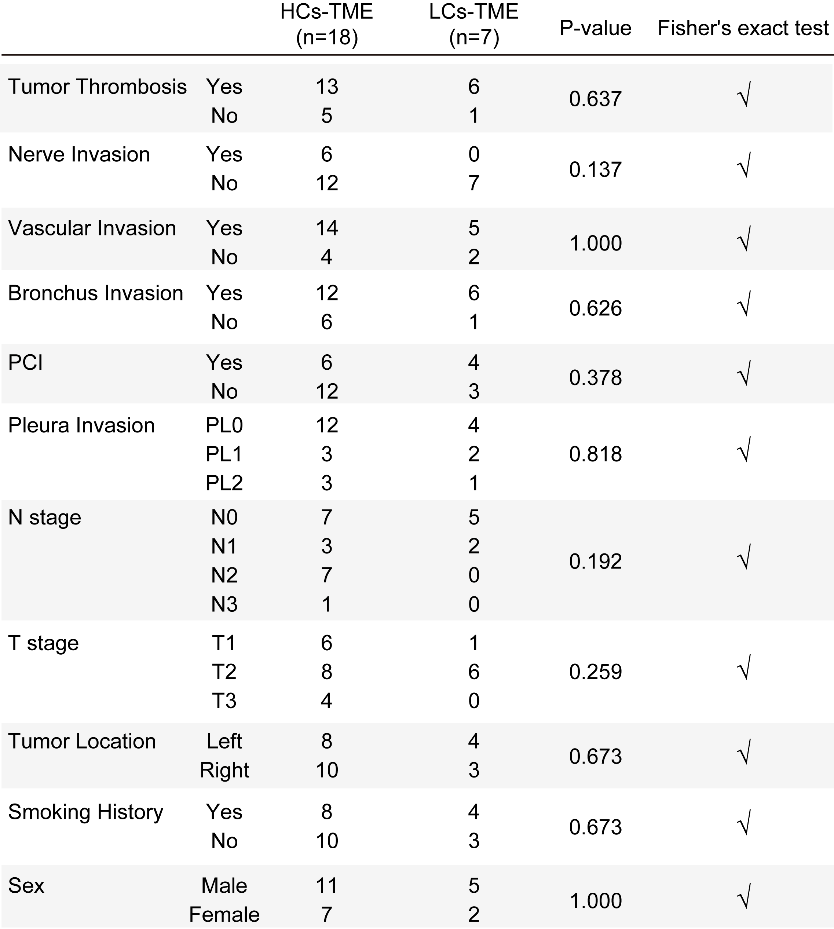
**

**Supplementary Figure 6. Distribution of clinicopathologic characteristics between HCs-TME phenotype and LCs-TME phenotype.** *P* values were calculated using Fisher’s exact test.

**Supplementary Table 1. The list of coefficient of variation (CV) for all RNAs (N=18,676).**

**Supplementary Table 2. The list of ITH scores for each ROI (N=79).**

**Supplementary Table 3. The list of spatial physical distance (SPD) between two ROIs on a single tumor.**

**Supplementary Table 4. The list of C-scores between two ROIs from the single tumor.**
